# Comprehensive Assessment of Anterior and Posterior Lingual Mandibular Depressions Using Cone Beam Computed Tomography: Morphology, Prevalence and Clinical Implications

**DOI:** 10.31661/gmj.v13iSP1.3703

**Published:** 2024-12-31

**Authors:** Seyyed Sajjad Pishva, Negar Sarrafan, Aisan Ghaznavi, Paria Fathi

**Affiliations:** ^1^ Department of Oral and Maxillofacial Medicine, School of Denticity, Urmia University of Medical Science, Urmia, Iran; ^2^ Department of Oral and Maxillofacial Radiology, School of Denticity, Urmia University of Medical Science, Urmia, Iran

**Keywords:** Cone-Beam Computed Tomography, Dental Implants, Lingual Subfossa, Mandible

## Abstract

Background: Assessment of lingual mandibular depressions, both anterior and posterior, has significant clinical relevance in various dental and maxillofacial procedures. Cone Beam Computed Tomography (CBCT) has emerged as a valuable tool for the detailed evaluation of these anatomical features due to its high-resolution imaging capabilities and three-dimensional visualization. The aim of this study was to comprehensively assess both anterior and posterior lingual mandibular depressions utilizing CBCT imaging, offering insights into their morphology, prevalence, and potential clinical implications. Materials and Methods: In this descriptive-cross-sectional study, 384 images from patients were examined. The images were reviewed using the Plunmeca Promax 3D device. In these images, the concavity depth, ridge thickness from the alveolar crest area, angle of concavity two millimeters above the inferior alveolar nerve, height of concavity from the start of the concavity to its end, and also the linear height along the occlusal plane with the opposing teeth in the lower jaw ridge were measured in the lingual area of the canine-premolar, first molar, and second molar. Based on the normal distribution of the data, parametric tests (Pearson correlation) were employed. According to the ICC, agreement between observers was estimated at 0.8. Results: The frequency of concavity was 2.9% in the canine-premolar region, in the first molar region 34.7%, and in the second molar region 98.2%. The concavity depth in the canine-premolar region was measured at 4.41 millimeters, in the first molar region at 3.80 millimeters, and the second molar region at 4.43 millimeters. The concavity height was reported as 13.26 millimeters in the canine-premolar region, 12.35 millimeters in the first molar region, and 13.51 millimeters in the second molar region. The angle of concavity was measured at 60.48 degrees in the canine-premolar region, 59.66 degrees in the first molar region, and 61.50 degrees in the second molar region. Ridge thickness in the canine-premolar region was 9.06 millimeters, in the first molar region 10.47 millimeters, and the second molar region 10.43 millimeters. No interference was found in the canine-premolar region, while interference was observed in 7.25% of cases in the first molar region and 23.6% in the second molar region. Additionally, a significant correlation was found between the concavity depth and interference with implants. Conclusion: Imaging with CBCT should be performed before implant placement also the concavity depth in the area should be considered to avoid potential interference during implant placement. This emphasizes the importance of thorough preoperative assessment for successful implant procedures.

## Introduction

Mandibular depressions, particularly anterior and posterior lingual depressions, are anatomical variations of the mandible that haveclinical significance in various dental and maxillofacial procedures. These depressions, often subtle and variable in presentation, can pose challenges in implant placement, endodontic treatments, and accurate diagnosis of pathologies [1]. The compressive force exerted by the submandibular and sublingual salivary glands on the bony cortex is a primary factor in the formation of these lingual depressions. Most perforations associated with implant placement occur in the submandibular fossa [2][3]. Although lingual plate perforation in the submandibular fossca may be asymptomatic, it can potentially damage the arteries in the sublingual region, leading to life-threatening hemorrhages [4][5][6]. Therefore, the proximity of critical anatomical structures, including the mandibular canal and mental foramen, necessitates thorough evaluation before implant surgery. Multiple factors shouldbe considered in the treatment planning phase before implant placement [2].

Periapical and panoramic radiographs have been used in implant treatment planning in the past. Although conventional radiographs are inexpensive and easily accessible, they have several inherent limitations, including magnification and distortion, the superimposition of adjacent structures, and the inability to provide buccolingual assessment [7]. Clinical palpation of the alveolar ridge offers limited information about the presence of lingual depressions [7][8]. Cone Beam Computed Tomography (CBCT) has revolutionized dental imaging by providing three-dimensional (3D) visualization of the maxillofacial structures with high precision and low radiation exposure [5][9]. Unlike traditional two-dimensional imaging techniques, CBCT offers detailed insights into the bony architecture of the mandible, allowing for more accurate identification and assessment of anatomical variations, including lingual depressions [8]. In the case report by Altwaim and Al-Sadhan, CBCT revealed bilateral anterior lingual depressions in the patient’s mandible. The depressions measured 2.1 cm wide and 0.59 cm deep on the right side, and 2.9 cm wide and 0.6 cm deep on the left side.10 CBCT mitigates the limitations of conventional radiography and clinical palpation by offering cross-sectional views and three-dimensional reconstruction of the mandibular structures [8][10].

The presence of undercuts in the lingual regions of the mandible makes this area a high-risk zone for implant placement. Unintentional perforation of the lingual cortex can lead to arterial injury and hematoma formation in the sublingual and submandibular regions, particularly in patients with atrophic ridges and proximity to the floor of the mouth [11][12].

Even though CBCT imaging has provided new insights intothe localization and morphological characteristics of lingual mandibular depressions most previousresearch investigations have been performed on samples non-Iranian origin. These studies have shown some distinctive morphologic variations in the mandible among different ethnic populations due to gene and environmental factors [13][14]. For instance, Nickenig et al. (2020) identified significant differences in the depth and angle or lingual concavities in European samples the findings of which may not generalize to the Iranian population [15]. Such differences of the populations emphasize the importance of regional research on the basis of the specific morphological characteristics, which may be significant for clinical work in various areas.

Furthermore, current studies highlight the use of CBCT imaging in the analysis of implant site risk factors most especially at areas that have deep lingual fosse [16][17]. Still, there is no special vigorous study concerned with the Iranian population, through which could be introduced beneficial information regarding to the rate of these concavities and their morphology. Thus, filling this gap, the present study will help to improve the safer and more effective treatment planning related to patients’ specific anatomical features in this area.

To prevent these issues, it is essential to be aware of the morphology, dimensions, and characteristics of the submandibular and sublingual fossae. Previous studies have demonstrated that the presence and characteristics of lingual depression can vary significantly among different populations. These variations can affect the outcomes of dental procedures, necessitating a tailored approach to treatment planning. However, there is limited research has focused on the Iranian population, which highlights the necessity of conducting localized studies to better understand these variations. Given that no studies have been conducted in this geographical region, this study aimed to investigate the prevalence and extent of lingual depressions’ interference with the implant pathway using CBCT.

## Materials and Methods

**Figure-1:**
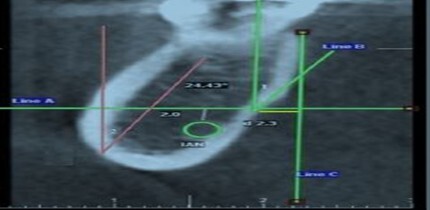
Figure 1. The angle between Line B and Line A as the Concavities Angle

**Figure-2:**
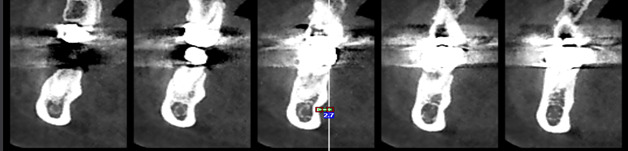
Figure 2. Depth of Concavities - Horizontal Distance between Point A and Lin

**Figure-3:**
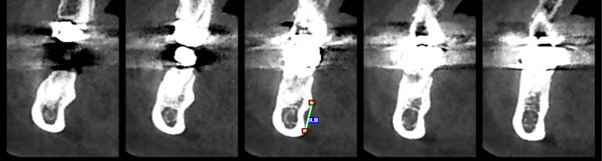
Figure 3. Measurement of Lingual and Mandibular Sublingual Concavities

**Figure-4:**
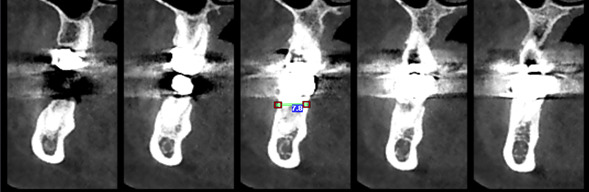
Figure 4. Measurement of Ridge Thickness in the Alveolar Crest Area

In this descriptive cross-sectional study, all male and female patients who visited private clinics in the city of Urmia, Iran, were included via convenience sampling, as data were sourced from archived CBCT images at private clinics. To achieve this, all archived data from the radiology offices of two specialty doctors, covering the years 2015 to 2017, were reviewed to obtain 384 cases. Although the study utilized historical data, the anatomical features assessed are unlikely to have undergone significant temporal changes because they are primarily determined by skeletal and genetic factors.

The sample size was determined using Cochran’s formula for an infinite population, as shown below:

n = (t^2^ pq) / d ^2^

The inclusion criteria for the sample were age above 18 years and complete visualization of the mandible. The exclusion criteria were the presence of artifacts in CBCT images making it difficult to identify reference points for measurement; patients with pathologies that severely affected the dimensions of the alveolar bone, including jaw diseases caused by metabolic, developmental, or inflammatory factors; patients with jaw fractures or who had undergone orthognathic surgery; and the presence of implants, grafts, or improper dental positions. The images were obtained using a Planmeca Promax 3D (Helsinki, Finland) machine with 12 mA, 90 kV, a duration of 12 seconds, and a voxel size of 0.2 millimeters. All measurements were performed by two observers (a specialist in oral and maxillofacial radiology and a periodontist), who were calibrated before the study began. The software used in this study was Planmeca Romexis 3.8.1. The areas examined in this study were the mandibular molar, premolar, and canine regions. First, brightness and contrast were adjusted, and then the angle of the mandibular plane relative to the horizontal line was corrected in the coronal and sagittal planes. Next, in the panoramic view, the position of the teeth adjacent to the area was aligned as vertically as possible to correct the angle of the mandibular plane relative to the horizontal line. Then, 2-millimeter-thick sections were selected in the desired areas. The sublingual and submandibular fossae were delineated.

The position of the mandibular canal was determined, and a horizontal line 2 millimeters above the canal in the selected section was identified (line A). Point A was the intersection of the lingual plate with line A. Line B was tangent to point A and parallel to the lingual plate. The angle between line B and line A was defined as the oncavities angle (Figure-1).

**Figure-5:**
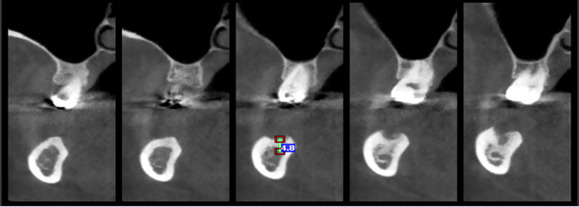
Figure 5. Relationship between Concavities Depth and Implant Interference

The concavities depth was the horizontal distance between point A and line C (line C: a line perpendicular to line A from the most prominent point of the buccal and lingual surfaces) (Figure-2). To assess the length of the lingual and mandibular sublingual concavity’s, the most prominent points above and below in the concavities area were identified in the sagittal section, and a line was drawn between them to measure their length (Figure-3). The ridge thickness in the alveolar crest area was measured. To determine the relationship between concavity depth and interference with implant placement and its prevalence, a line representing implant placement in the occlusion path with opposing teeth in the lower jaw ridge was drawn (Figure-4). This relationship was examined by the interference of this line with the Concavities (the implant used in this study was a standard implant with a length of 8 millimeters) (Figure-5). For assessing operator reliability, 10% of the samples were randomly selected, and all variables were remeasured. The interval between the two assessments was two weeks. ICC (Intraclass Correlation Coefficient) was used to examine both inter- and intra-reliability. The data were entered into IBM SPSS Statistics for Windows, version 19 (IBM Corp., Armonk, N.Y., USA) for analysis after collection.

Descriptive statistics (mean and standard deviation) and parametric tests (Pearson correlation) based on the normal distribution of the data were employed. According to the ICC, agreement between observers was estimated at 0.8. The significance level of the data in this study was considered P < 0.05.

## Results

**Table:** Table1. Mean and standard deviation of Concavity Depth, Concavity Height, Concavity Angle, Ridge Thickness, and Implant Placement Interference in the Premolar Canine, First Molar, and Second Molar Regions

Parameter	Concavities Depth Mean ± SD	Concavities Height Mean ± SD	Concavities Angle Mean ± SD	Ridge Thickness Mean ± SD	Implant Placement Interference Mean ± SD
**Premolar Canine**	4.41±0.99	13.26±2.53	60.48±6.90	9.06±1.12	-
**First Molar**	3.8±1.11	12.35±1.81	59.66±8.00	10.47±8.60	5.78±1.93
**Second Molar Regions**	4.43±1.31	13.51±1.95	61.50±23.46	10.43±2.08	7.01±0.77

**Table:** Table2. Pearson Correlation Test of Concavities Depth with Implant Placement Interference in the First and Second Molar Regions

**Parameter**		**Interference in the First Molar**	**Interference in the Second Molar **
	Pearson Correlation Coefficient	3.51	1.36
Concavities Depth in the First Molar	Sig.	0.001	0.004
	Number	18	51
	Pearson Correlation Coefficient	2.30	1.51
Concavities Depth in the Second Molar	Sig.	0.001	0.007
	Number	31	183

Out of 384 cases, 11 (2.9%) exhibited concavities in the premolar canine region, while 133 cases (34.7%) showed concavities in the first molar region, and 377 cases (98.0%) displayed concavities in the second molar region. No instances of implant placement interference were reported in the premolar canine region; thus, statistical analysis for this area is unavailable. Radiographic examination revealed that 10 cases (7.5%) out of 133 in the first molar region and 89 cases (23.6%) out of 377 in the second molar region exhibited interference (Table-1).

The results revealed that the highest mean concavities depth, concavity, height, and concavity angle was associated with the second molar region, followed by the premolar canine and first molar regions in the second to third ranks. Additionally, the highest mean ridge thickness was observed in the first molar region, followed by the second molar and premolar canine regions in the second to third ranks. Based on the information provided, no interference was reported in the premolar canine region. Therefore, the correlation coefficient is calculated only for the concavity depth and the level of interference in the first and second molar regions. The results indicated a significant correlation between concavity depth and interference, showing that as the concavity depth increases, the interference also increases (Table-2).

## Discussion

The present study aimed to investigate the anterior and posterior lingual mandibular tori using cone beam computed tomography. In this study, the prevalence of tori in the premolar region was 9.2%. Tori prevalence in the first molar region was 7.34%, and in the second molar region was 2.98%.

In a study by Nickenig et al., the prevalence of concavities in the premolar region was measured at 4.14%, and in the molar region it was 7.68% (without distinguishing between the first and second molars) [18]. In other studies, the lingual aspect of the first molar has been examined. In the study of Chan et al. [19], the prevalence of concavities was 66%, Panjnoush et al. [15] found it 56%, and Herranz Aparicio et al. [16] revealed 64.2%. The reason for the differences in results could be because of the first and second molars and premolar canines that were not separated from each other. Additionally, in a study by Nickenig et al., differences in race and failure to distinguish between the first and second molars in the molar region could also be contributing factors.

In this study, the depth of concavities in the premolar region was measured at 4.14 millimeters, in the first molar region at 3.80 millimeters, and in the second molar region at 4.43 millimeters. In the study of Nickenig et al. [18], the depth of tori in the premolar-canine region was 0.80 millimeters, and in the molar region, it was 3.70 millimeters. In another study by Panjnoush et al. [15], the depth of lingual mandibular concavities in the first molar region was measured at 1.30 ± 1.54 millimeters. In a study by Chan et al. [19], it was 2.4 millimeters, and in the study of Herranz Aparicio et al. [16], it was 3.6 millimeters. The differences in the obtained measurements could be due to racial differences, since Chan et al. focused on individuals of African descent in their study, or methodological variations, as seen in a study by Panjnoush et al. Unlike all the reviewed articles, the method used to measure the height of lingual mandibular concavities considered the end of the torus as the endpoint of torus height instead of the mandibular sublingual border. According to the results, the height of tori in the premolar region was 13.26 millimeters, the first molar region was 12.35 millimeters, and the second molar region was 13.51 millimeters. However, the height of tori was found to be 20.3 millimeters by Herranz Aparicio et al. [16]; in a study by Chan et al. [14], it was 14.9 millimeters, and in a study by Nickenig et al. [18], it was 29.1 millimeters in the premolar-canine region and 24.9 millimeters in the molar region. As mentioned earlier, variations in measurement methods and racial differences could be the reason for the difference in results.

In this study, the angle of concavities in the premolar region was measured at 60.48 degrees, in the first molar region at 59.66 degrees, and in the second molar region at 61.50 degrees. In a study by Nickenig et al. [18], the angle of concavities in the premolar region was 85.7 degrees, and in the molar region, it was 53.9 degrees. Additionally, in the Panjnoush et al. study [15], the angle of concavities was reported as 16.19 ± 15.45 degrees; Herranz Aparicio et al. [16] reported 69.5 degrees, and Chan et al. [9] revealed 57.7 degrees. In these studies, differences in the obtained numbers may arise from variations in measurement methods and the small sample size (Panjnoush) as well as racial differences. In similar studies, the measurement method considered the thickness of the ridge 2 millimeters below the alveolar crest, whereas, in this study, measurements were taken from the crest itself. The ridge thickness in this study was measured in the premolar-canine region; it was 9.06 millimeters; in the first molar region, it was 10.47 millimeters, and in the second molar region, it was 10.43 millimeters. Chan et al. [19] reported the ridge thickness as 2.7 millimeters; in the Herranz Aparicio et al. study, [16] it was 10.1 millimeters; and in the Nickenig et al. study [18], it was 6.7 millimeters in the premolar-canine region and 7.9 millimeters in the molar region. The reasons for the differences in the obtained numbers could be attributed to differences in measurement methods and racial disparities.

This study was the first to investigate the prevalence of implant interference with concavitydepth, and we didn’t find similar articles in this field. In this research, a linear height, extending from occlusion with opposing teeth in the lower jaw ridge to the beginning of the torus, should exceed 8 millimeters; otherwise, it was considered interference. In the premolar-canine region, no interference was observed. In the first molar region, 10 out of 133 cases (7.25%) exhibited interference, while in the mandibular molar region, 89 out of 377 cases (23.6%) showed interference with implants. As the depth of the torus increases, interference with implants also increases. The presence of undercuts in the lingual mandibular areas makes this region particularly vulnerable during implant placement. Accidental disruption of the lingual cortex can lead to arterial damage and hematoma formation in the sublingual and submandibular regions, especially in patients with atrophic ridges and proximity to the oral floor, exacerbating the situation [18].

Limitations and sugestions

This study’s limitations include examining a limited local population over two years, potentially compromising the generalizability of findings. The retrospective nature of the study poses challenges in establishing causality and tracking changes over time due to reliance on existing data. Utilizing a single CBCT machine may restrict the study’s relevance to other systems with differing capabilities.

Suggestions for future research involve expanding sample sizes across various demographics, conducting longitudinal studies to assess long-term impacts, and correlating morphological data with clinical outcomes to enhance the understanding of lingual mandibular depressions in dental procedures.

## Conclusion

Evaluation of the mandibular ridge using CBC before implant placement provides dentists with the opportunity to accurately assess and evaluate the topography of the ridge before implant placement, ensuring precise and proper implant placement. Furthermore, the significant correlation between the depth of tori and implant interference in the lingual mandibular region underscores the importance of considering this correlation before implant placement. Attention to this aspect can help prevent potential complications during implant placement and ensure successful outcomes for patients undergoing implant procedures. Therefore, incorporating CBCT evaluation into the preoperative assessment protocol can significantly contribute to the success and safety of implant dentistry practice.

What is current knowledge?

Previous research has focused on the anatomical characteristics of mandibular bone structures using imaging techniques like conventional radiography, panoramic radiography, and initial CBCT studies. These studies have described the general morphology, prevalence, and variations of mandibular concavities. They have also quantified the prevalence and distribution of lingual concavities in different populations, noting variability based on demographic factors such as age, gender, and ethnicity. Additionally, research has compared the efficacy of various imaging modalities in identifying and assessing mandibular concavities, highlighting the shift from 2D to 3D imaging with the advent of CBCT.

What is New Here?

This study uniquely provides a comprehensive assessment of both anterior and posterior lingual mandibular depressions using CBCT imaging. It offers detailed measurements of concavity depth, ridge thickness, angle of concavity, and other parameters in specific regions of the mandible, providing insights into morphology, prevalence, and clinical implications. The study also establishes a significant correlation between concavity depth and implant interference, underscoring the importance of preoperative CBCT imaging for successful implant procedures.

## Acknowledgment

The authors appreciate and thank the patients participating in this study. This work was supported by the Deputy for Research of Urmia University of Medical Sciences.

## Conflict of interest

None.
